# Expect the Unexpected: Torsion of the Gallbladder, a Rare Cause for Acute Cholecystitis

**DOI:** 10.7759/cureus.3726

**Published:** 2018-12-13

**Authors:** Yang Hwang, Krish Kulendran, Jack Ashworth

**Affiliations:** 1 Surgery, Redland Hospital, Brisbane, AUS

**Keywords:** gallbladder, torsion, volvulus, elderly, cholecystitis

## Abstract

Gallbladder torsion is a rare cause for acute cholecystitis. Gallbladder torsion is unlikely to respond to conservative management and requires urgent surgical intervention. We report a case of an 85-year-old female with gallbladder torsion. She presented with a clinical picture consistent with acute cholecystitis. Radiological findings showed signs that elude to the diagnosis and intra-operative findings showed a complete torsion with a free-floating gallbladder. This case highlights the need to have a high index of suspicion for gallbladder torsion as a differential diagnosis for right upper quadrant (RUQ) pain apart from the typical calculous acute cholecystitis, especially in high risk groups such as thin, elderly females. Prompt decision for cholecystectomy is necessary and is likely to result in a good outcome.

## Introduction

The “floating gallbladder” was first described by Wendel in 1898 [1], since then over 500 cases have been reported, with 105 of these identified laparoscopically [2]. Gallbladder torsion is predominantly a condition of the elderly affecting those between the ages of 60 and 80, however, children can also be affected [3]. There is an incidence of one in 365,520 hospital admissions with a female preponderance ratio of 3:1.4. It carries an associated mortality of 6% [2].

The most commonly accepted mechanisms for gallbladder torsion in adults are mainly due to the two mesenteric anatomical variations. These include a long mesentery allowing the gallbladder and its associated cystic vessels to rotate along its axis, otherwise known as the “floating or pedunculated” gallbladder. In elderly patients, a loss of visceral fat combined with liver atrophy can result in an acquired long mesentery. Rarer cases have been described where kyphoscoliosis and atherosclerosis of the cystic artery have played a role [4].

Gallbladder torsion can be classified as either complete (>180 degree) or incomplete (<180 degree). The rotation that occurs can be both clockwise or counter-clockwise, and the direction of rotation is attributed to the source of hyper-peristalsis. A clockwise rotation of the gallbladder pedicle is thought to be caused by gastric hyperperistalsis, whereas counter-clockwise with colonic [2]. The torsion directly affects the blood supply to the gallbladder causing infarction and necrosis.

Patients present with sudden acute pain in the right upper quadrant (RUQ), and a story suspicious for acute cholecystitis. Imaging may show changes consistent with cholecystitis. In addition to gallbladder wall thickening and pericholecystic fluid, indicators of gallbladder torsion include a low-lying gallbladder below its usual anatomical fossa, conical or V shaped gallbladder, whirl sign of the cystic artery or bulls eye sign on hydroxyiminodiacetic acid (HIDA) scan [2]. Kitagawa et al. proposed four computed tomography (CT) diagnostic criteria for gallbladder torsion: an abnormal horizontal axis to the gallbladder rather than the expected vertical orientation within the gallbladder fossa, a fluid collection in the gallbladder fossa, presence of well-enhanced cystic duct on right-hand side of gallbladder, gallbladder wall thickening [5]. The presence of gallstones is described in only 36% of patients with torsion [2].

The following case report is a typical presentation of gallbladder torsion, with supportive imaging and intra-operative images. This case report highlights clinical features that help diagnose this rare condition which requires prompt surgical intervention.

## Case presentation

**Figure 1:**
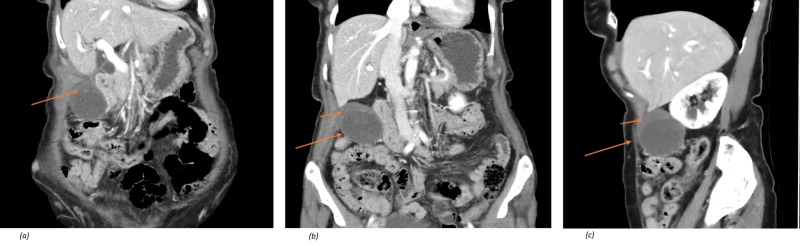
Computed tomography (CT) of torted gallbladder. (a) V-shape superior portion of gallbladder with asymmetrical thickening of gallbladder wall. (b) Low and horizontal lying of gallbladder. (c) Gallbladder lower than inferior liver edge, rather than typical anatomical position within gallbladder fossa. Short arrows - Hypoattenuation of the gallbladder wall compared with surrounding visceral structures.

A thin (41 kg) 85-year-old female presented with a 10-hour history of severe RUQ pain, nausea and vomiting. Her background included multiple lower abdominal laparotomies for gynaecological procedures, hypertension and dyslipidaemia. There were no clinical features of systemic upset. Her examination revealed a tender and guarded RUQ. Liver function tests were normal, white cell count was mildly elevated. Ultrasound demonstrated a distended gallbladder, without cholelithiasis and an asymmetrical gallbladder wall thickening to 8.5 mm. CT scan confirmed the diagnosis of acalculous cholecystitis, V-shaped superior portion of the gallbladder, low and horizontal lying gallbladder with hypoattenuation of the gallbladder wall compared with surrounding visceral structures (Figures 1a-1c). After 24 hours of observation whilst on broad-spectrum antibiotics and simultaneous fluid resuscitation, the patient failed to clinically improve. The decision was made to undergo laparoscopic cholecystectomy.

**Figure 2:**
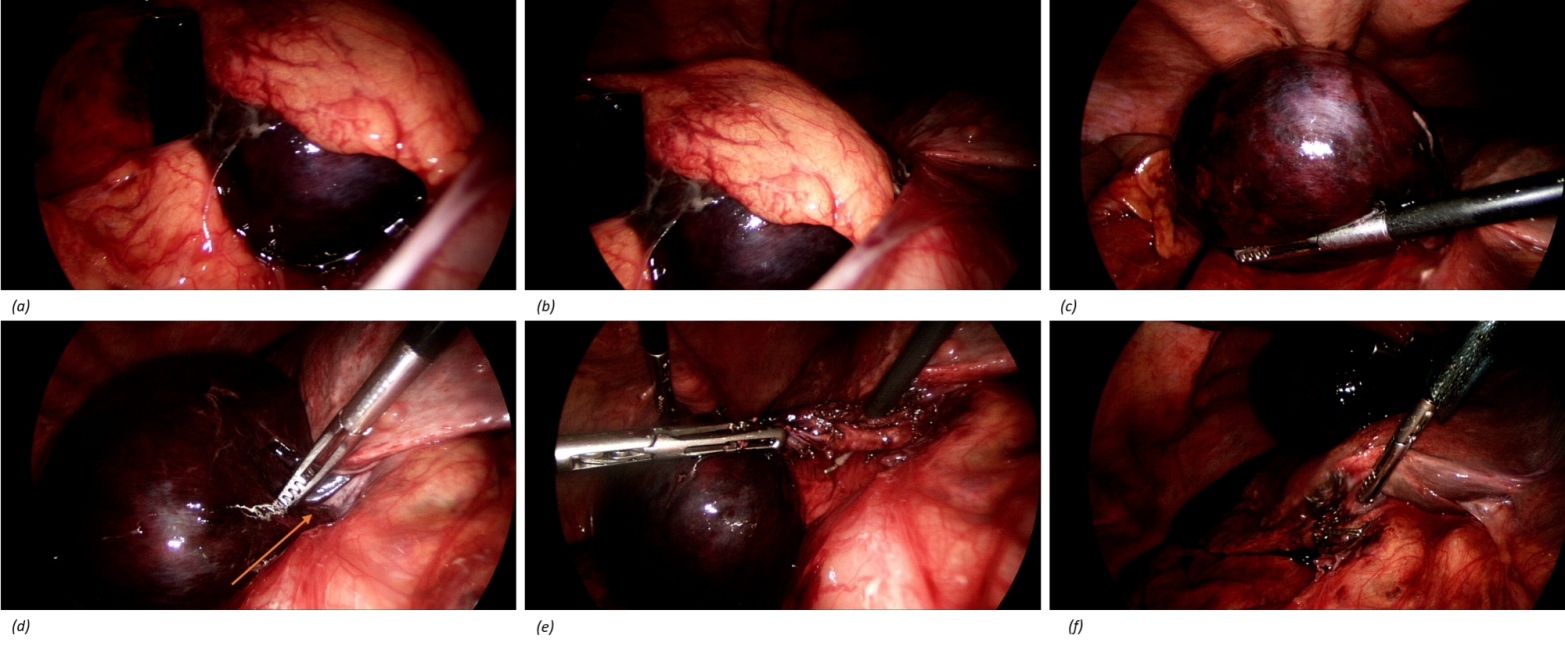
Intra-operative findings of torted gallbladder. (a, b) Gangrenous gallbladder with omental adhesions. (c) Floating gallbladder within abdominal cavity. (d) Floating gallbladder on pedicle consisting of cystic duct and cystic artery. (e) Detorsion of gallbladder and display of pedicle. (f) Cystic duct and artery clipped and cut at the completion of cholecystectomy.

Intra-operative findings revealed a large, necrotic, completely torted and floating gallbladder. Its only attachment appeared to be the cystic duct and cystic artery on which the gallbladder had twisted 360 degrees in an anti-clockwise direction (Figures 2c-2d). Principles of the operation include derotation then cholecystectomy and intra-operative cholangiogram. To establish the required critical view, it was necessary to unravel the torted pedicle before proceeding with the dissection. The cholecystectomy was otherwise routine. Gallbladder was retrieved in an endoscopic specimen retrieval bag and decompressed in the bag to allow delivery of the specimen and prevent spillage of bile intra-abdominally.

Our patient’s discharge was delayed by an asymptomatic demand-related cardiac ischaemia, requiring 24-hour period of observation. She was discharged day two post-operatively with a plan for cardiology follow-up. Histology showed acute gangrenous cholecystitis.

## Discussion

Although there are a wide range of imaging modalities available to make a pre-operative diagnosis, they are not essential. Clinical findings and a high index of suspicion remain the most useful tool in the decision to proceed to cholecystectomy [6]. Our patient was an elderly, frail female with a presentation and ultrasound suspicious for acute acalculous cholecystitis, who had poor response to broad spectrum antibiotics. Although we did not make the pre-operative diagnosis of gallbladder torsion, we recognized that this patient was unlikely to resolve with conservative management and would not likely survive gallbladder perforation and biliary peritonitis.

## Conclusions

This case highlights the need to have a high index of suspicion for gallbladder torsion as a differential diagnosis for RUQ pain apart from the typical calculous acute cholecystitis, especially in high-risk groups such as thin, elderly females. Prompt decision for cholecystectomy is necessary and is likely to result in a good outcome.
